# The effect of parental and teacher autonomy support and core self-evaluations: a three-wave longitudinal study of middle students’ career adaptability

**DOI:** 10.3389/fpsyg.2024.1404478

**Published:** 2024-09-30

**Authors:** Xiaoyun Zhao, Shun Huang, Chaofan Shi

**Affiliations:** ^1^College of Education, Huaibei Normal University, Huaibei, China; ^2^Academic Affairs Office, Huaiyin Normal University, Huai’an, China

**Keywords:** career adaptability, core self-evaluations, parental autonomy support, teacher autonomy support, cross-lag model

## Abstract

**Introduction:**

According to career construction theory, middle school students are in a critical phase of growth and exploration that requires self-reflection on their interests, values, and aspirations. Career adaptability is a key indicator of career development for middle school students and a crucial ability for achieving career success. Research indicates that many Chinese middle school students face significant hurdles in their career development, including a lack of motivation, limited self-awareness, and unclear career trajectories.

**Objective:**

To address these challenges, it is imperative to explore the factors influencing career adaptability, with a particular focus on the role of parental and teacher autonomy support within the framework of self-determination theory. This study aims to explore the correlation between parental autonomy support (PAS), teacher autonomy support (TAS), core self-evaluations (CSE), and career adaptability (CA) among middle school students.

**Methodology:**

The longitudinal data for this study were collected from two middle schools in the Anhui province. Middle school students were recruited as research participants through a cluster sampling method. A total of 482 students were surveyed in three stages during a 1-year period, and a cross-lag model was employed to analyze the data.

**Results:**

(1) From T1 to T2, T1PAS predicted T2CS, T1CSE predicted T2CA, and T1CA predicted T2TA; (2) from T2 to T3, T2PAS predicted T3CS, T2CSE predicted T3CA, and T2TAS predicted T3PAS; (3) TAS did not predict CSE and CA over time; (4) T2CSE mediated the relationship between T1PAS and T3CA.

**Discussion:**

These findings suggest that autonomy support has a consistently positive influence on the career development of early adolescents. Valuing children’s autonomy is beneficial for fostering positive self-evaluations and shaping their career trajectories.

**Conclusion:**

Autonomy support plays a pivotal role in enhancing middle school students’ career adaptability and promoting career development by strengthening self-evaluations. Additionally, the effect of parental autonomy support is more stable than that of teacher autonomy support.

## 1 Introduction

Career development is a lifelong journey and a pivotal task for middle school students. According to career construction theory, adolescents are in the growth and exploration phase of their career development (Karacan-Ozdemir and Ayaz, 2022). At this stage, middle school students focus on reflecting on interests and values, developing self-identity, and gaining knowledge about career planning, all of which help them make informed decisions regarding their future career paths (Yuen and Yau, 2015).

The career development of Chinese middle school students faces numerous challenges. First, many middle school students have low career confidence and self-awareness., which results in confusion when choosing study paths and planning careers due to a limited understanding of their interests and strengths (Wang et al., 2024). They struggle to set clear and achievable short-term and long-term goals, which hinders their ability to develop sound plans for their future (Hu et al., 2024).

Second, parental autonomy support for middle school students is often lacking. Many parents focus too much on their children’s academic performance and admission to prestigious schools, neglecting their personal development and career interests (Chen et al., 2021).

This dynamic increases students’ stress, discouraging them from pursuing their interests.

Additionally, communication between parents and children regarding future development is often inadequate, leaving students without essential guidance when choosing their academic and career paths (Liang et al., 2022).

Third, there are significant shortcomings in career education at the middle school. The career education courses provided by schools often lack a structured and practical approach to effectively guide students in career planning (Xie et al., 2019). Additionally, there are limited opportunities for career exploration and extracurricular experiences (Gu et al., 2020), making it difficult for students to gain firsthand knowledge of various professions. To some extent, the above situation May lead to the low career adaptability observed among Chinese middle school students (Chen et al., 2020).

Career adaptability is a core indicator of adolescent career development, reflecting the psychological resources needed to navigate careers and a key capability for achieving career success (Brown and Lent, 2016; Cristy and Kurniawati, 2023). Career adaptability helps middle school students set realistic goals and make informed career decisions.

Research shows that Chinese middle school students face numerous challenges in their career development, including inadequate motivation for learning, limited self-awareness, and unclear understanding of future career paths (Wang et al., 2024). Hence, it is imperative to investigate the influencing factors and mechanisms shaping middle school students’ career adaptability.

Career construction theory posits that individual career development is a dynamic process of mutual adaptation between the inner self and the external environment. The content and results of this construction vary from person to person (Savickas, 2020). In essence, career development should reflect a process of self-determination and self-adjustment.

When young people proactively pursue their career paths, it is essential for parents and teachers to provide them with autonomy support to foster their career advancement. However, in China, young individuals are confronted with intense competitive pressures from an early age, such as high school and college entrance examinations (Sun et al., 2012).

Parents and educators often adopt an authoritative approach, overly interfering with teenagers’ growth and limiting their autonomy (Lu and Chang, 2013). Therefore, it is imperative to investigate the impact of autonomy support from parents and teachers on the vocational adaptation of middle school students within the context of the self-determination theory in China.

Autonomy support acts as an external environmental factor, while career adaptability and core self-evaluations—representing internal psychological resources and self-perception—serve as critical links between autonomy support and career adaptability. Essentially, students who receive greater autonomy support are more likely to develop positive self-evaluations and are better equipped to create a harmonious adaptation between their inner selves and the external environment (Neubauer et al., 2021).

Currently, research on the relationship between parental and teacher autonomy support and career adaptability in China is relatively superficial, with a greater focus on social support and less on autonomy support.

This study aims to examine the impacts of parental and teacher autonomy support, along with core self-evaluations, on middle school students’ career adaptability through longitudinal tracking and analysis of each variable’s mechanisms.

This study seeks to clarify the relationship between autonomy support and career adaptation, its underlying psychological mechanisms, and to contribute to both theoretical research and practical applications in middle school students’ career development. Furthermore, it aims to encourage educators to provide career guidance, inspire parents and teachers to provide more autonomy support, and help students develop clear career plans.

### 1.1 Parental and teacher autonomy support and career adaptability

Family and school serve as micro-systems that influence students’ growth, with parents and teachers playing crucial roles as significant figures in students’ developmental journey, directly impacting their progress (Jeon et al., 2021).

According to career construction theory, students’ career development is shaped by a combination of individual and environmental factors (Johnston, 2018).

Environmental factors such as schools (including teacher support) and families (through parental support) play key roles in shaping students’ career development (Xiao et al., 2021).

Simultaneously, the rise of positive psychology has highlighted self-determination theory, encouraging scholars to focus on the autonomy support provided by parents and teachers (Grolnick and Pomerantz, 2009). In line with the self-determination theory, an autonomous environment fosters individuals’ self-exploration, self-discovery, and self-understanding.

With external support, they can develop their emotional and value systems, advancing their career development (Gagné et al., 2022).

According to Deci and Ryan (2012), parental autonomy support entails parents respecting their children’s opinions, offering opportunities for autonomous decision-making, refraining from verbal control, fostering an atmosphere of autonomy within the family, and encouraging the autonomy growth of their children. Mageau and Vallerand (2003) posit that teacher autonomy support involves students perceiving the support and respect extended by teachers, fostering students’ autonomy development, nurturing students’ capacity for independent thinking, and offering ample information to explore and make choices autonomously. Middle school students are in a critical period of career exploration, and their career development is closely linked to the fulfillment of their autonomy needs.

During this phase, if parents and teachers create an environment that encourages and respects students’ choices, it will help them explore their interests and achieve career success. Tang et al. (2013) asserted that parental and teacher autonomy support can positively forecast the career development of middle school students. Additionally, Ataç et al. (2018) conducted a questionnaire survey on adolescents and discovered that the greater the social support, the higher the level of career adaptability.

This finding supports stage-environment fit theory, which posits that individuals can only achieve optimal development when their psychological needs are adequately met by external resources (Calzo et al., 2020; Eccles et al., 1993).

Parental and teacher autonomy support plays a vital role in meeting the evolving psychological needs of middle school students. Satisfying these psychological needs can stimulate internal motivation, which, in turn, impacts their career development. As key figures, parents and teachers have a profound influence on the career development trajectory of middle school students. Therefore, the following hypotheses are proposed:

*H1*: Parental and teacher autonomy support can positively predict middle school students’ career adaptability.

### 1.2 Parental and teacher autonomy support and core self-evaluations

Drawing from philosophy and clinical psychology, Judge et al. (1998) proposed the concept of “core self-evaluations,” which include self-esteem, generalized self-efficacy, locus of control, and emotional stability (non-neuroticism). Core self-evaluation is considered a key trait that affects an individual’s psychological processes, serving as the foundation for how one assesses their own abilities and value (Judge et al., 2003). The development of core self-evaluations is closely related to important figures, personal achievements, and other factors.

According to self-determination theory, parents and teachers, as important figures, can cultivate a nurturing and supportive environment that fosters understanding, encourages and respects individual choices, and actively guides individuals in self-exploration, which, in turn, helps individuals gain an accurate understanding of themselves and develop positive self-evaluations (Gagné et al., 2022). In their research on parental autonomy support and school adaptation among middle school students, Bouffard and Labranche (2023) discovered that the more support middle school students perceived from their parents, the more autonomy they were able to develop.

During autonomous decision-making, middle school students can develop a sense of capability and self-worth, which fosters self-confidence and positively influences their school adaptation and career development. Their recognition of parental and teacher autonomy support likely strengthens their core self-evaluations. Therefore, based on theoretical analysis and empirical research, the following hypotheses are proposed:

*H2*: Parental and teacher autonomy support can positively predict middle school students’ core self-evaluations.

### 1.3 Core self-evaluations and career adaptability

Career construction theory emphasizes that individuals can respond to changes in the career environment with positive attitudes and behaviors (Tak et al., 2015). In the career construction theory model, the individual adaptation process can be interpreted in the order of adaptation preparation, adaptation resources, adaptation response, and adaptation results (Savickas, 2020). As a typical measure of adaptation preparation, core self-evaluations are described as an individual’s comprehensive assessment of their own abilities and values (Hirschi et al., 2015). Adaptive resources refer to the psychosocial resources that individuals have to cope with changes, which is the CTT model’s key component and embodied in career adaptability. Individuals with a higher level of core self-evaluations have a more objective recognition of themselves, and those with a clearer self-awareness are likely to exhibit greater confidence in navigating career challenges and attaining career objectives, thus enhancing their level of career adaptability (Chen et al., 2001). The research by Zacher (2016) and Xu and Yu (2019) has revealed that core self-evaluations can positively forecast individual career adaptability. Based on theoretical analysis and empirical evidence above, the following hypotheses are proposed:

*H3*: Core self-evaluations can positively predict middle school students’ career adaptability.

*H4:* Core self-evaluations could play a mediating role between parental and teacher autonomy support for middle school students’ career adaptability.

## 2 Research method

### 2.1 Research design

This study is a quantitative longitudinal study using a longitudinal cross-hysteresis design study. The study design is particularly suitable for looking at causality and time series data. We used questionnaires to measure relevant variables at multiple time points (T1, T2, T3), and the interval was 6 months. Through statistical analysis of the structural equation model, a longitudinal cross-lag model was constructed to explore the relationship between parental and teacher autonomy support, core self-evaluations, and career adaptability.

### 2.2 Population and sample

The longitudinal data of this study were gathered from two middle schools in Anhui. Middle school students were selected as the research subjects using a cluster sampling method. A total of 578 participants were recruited in the initial experiment, followed by subsequent experiments conducted every 6 months, three experiments in total. Ultimately, 482 valid subjects were obtained, resulting in an overall attrition rate of 16.61%.

The inclusion criteria for the sample were as follows: (1) Participants must be aged between 10 and 18 years. (2) Participants must currently be attending middle school. (3) Participants must possess adequate language comprehension skills to ensure they can accurately understand and respond to the questions in the questionnaire. (4) Participants must receive sufficient information about the study’s purpose and involvement, and written consent must be obtained from their legal guardian or parent. (5) Participants must not have received any interventions or treatments related to the study topic, such as career counseling or psychotherapy. (6) Participants must have enough time to engage in the questionnaire survey and complete it in three designated periods.

Analysis of the differences in scores between the lost and retained subjects indicated that there were no significant discrepancies in career adaptability (*t* = 0.83, *p* = 0.41), core self-evaluations (*t* = −0.65, *p* = 0.52), parental autonomy support (*t* = 1.12, *p* = 0.26), and teacher autonomy support (*t* = 0.39, *p* = 0.69) at the first test. This suggests that there was no systematic loss of subjects. The average age of the subjects was 12.92 ± 0.64, with 292 boys accounting for 60.58% and 190 girls accounting for 39.42%.

### 2.3 Technique and instrument

A questionnaire survey was used to collect research data. We have obtained the informed consent of students and their legal guardians to inform them that the purpose of the study is to promote student career development. Students are required to fill out questionnaires on-site and can withdraw from the test at any time. Students are paid when they complete the test. If there is any negative impact during the test, we will immediately stop the test and provide appropriate psychological assistance and compensation. After data collection, we number the subjects, process the data anonymously, and prevent unauthorized access. The scale used in this study is as follows:

(1) Career Adapt-Ability Scale

The Career Adapt-Ability Scale developed by Savickas and Porfeli (2012) was used in this study, which consisted of four dimensions of attention, control, curiosity, and confidence, with six items in each dimension and a total of 24 items. Likert 5 points were used to score, with 1–5 representing not strong to very strong, and the smaller the number, the weaker the degree of agreement between the statement of the scale items and their own situation. The higher the total score on the scale, the stronger the individual’s career adaptability. At the T1, the Cronbach’s *α* coefficient of the scale was 0.87, and the subscales were between 0.72 and 0.78. At T2, the Cronbach’s *α* coefficient of the scale was 0.88, and the subscales were between 0.73 and 0.79. The Cronbach’s α coefficient of the scale at T3 was 0.92, and the subscales ranged from 0.75 to 0.80. (Note: T1 is short for time 1, which is the time when the subject first took the test. T2 is short for time 2, and T3 is short for time 3, the same as below.)

(2) Core Self-Evaluations Scale

The Core Self-Evaluations Scale was used by Judge et al. (2003). The scale consisted of 10 items, single dimension, and was scored by Likert-5 points, with 1 to 5 representing completely disagree to agree strongly. The second, third, fifth, seventh, eighth, and tenth items in the scale should be converted into reverse scoring items, and then the total average score should be calculated. The higher the score, the higher the level of core self-evaluations of middle school students. The Cronbach’s *α* coefficient of the scale at T1 was 0.77. The Cronbach’s α coefficient of the scale at T2 was 0.76. The Cronbach’s α coefficient of the scale at T3 was 0.78.

(3) Parental Autonomy Support Scale

The Parental Autonomy Support Scale revised by Wang et al. (2007) was used. The scale consisted of 12 items, single dimension, and was scored by Likert-5 points, with higher scores indicating more parental autonomy support perceived by children. The Cronbach’s α coefficient of the scale at T1 was 0.74. The Cronbach’s α coefficient of the scale at T2 was 0.87. The Cronbach’s α coefficient of the scale at T3 was 0.85.

(4) Teacher Autonomy Support Scale

The Teacher Autonomy Support Scale developed by Williams and Deci (1996) was used. The scale consists of 14 items, is single-dimensional, and is scored by Likert-5 points, with higher scores indicating more teacher autonomy support perceived by middle school students. In the longitudinal follow-up data, the Cronbach’s α coefficient of the scale at T1 was 0.81. The Cronbach’s α coefficient of the scale at T2 was 0.89. The Cronbach’s α coefficient of the scale at T3 was 0.85.

### 2.4 Data analysis

This study used SPSS 22.0 for data collection and statistical analysis. The data of participants who did not participate in all three surveys and admitted that they did not answer carefully or answered regularly, were deleted. Mplus 8.0 was used for confirmatory factor analysis and cross-lag model analysis.

### 2.5 Ethical considerations

We have obtained the informed consent of students and their legal guardians. First, we ensure that participating students and their legal guardians are aware of the study’s purposes, methods, and risks, voluntarily participate, and can withdraw from the study at any time. Second, we protect students’ personal information by anonymizing the data through numbering. Third, we take the necessary measures to ensure data security and prevent unauthorized access. Finally, given the psychological impact of research on students, we ensure that research does not unnecessarily negatively impact students. Take proactive measures to mitigate or eliminate negative effects and provide appropriate compensation when they occur.

## 3 Results

### 3.1 Test of common method bias

This study performed a common method deviation test on all questions in the Career Adaptability Scale, Core Self-Evaluations Scale, Parental Autonomy Support Scale, and Teacher Autonomy Support Scale by Harman single factor test. The results showed that the variance explained by the first common factor in the three measurements was 15.96, 20.01, and 20.70%, respectively, which was lower than the critical value of 40%, indicating no serious common method bias in this study.

### 3.2 Descriptive statistics and correlation analysis

Table 1 shows the mean, standard deviation, and correlation analysis results of the variables involved in this study. There were significant positive correlations among career adaptability, core self-evaluations, parental autonomy support, and teacher autonomy support at the three-time points.

**Table 1:** Descriptive statistics and correlation coefficient matrix (*N* = 482).

	1	2	3	4	5	6	7	8	9	10	11	12
1. T1CA	1											
2. T2CA	0.38^***^	1										
3. T3CA	0.34^***^	0.32^***^	1									
4. T1CSE	0.32^***^	0.23^***^	0.24^***^	1								
5. T2CSE	0.21^***^	0.42^***^	0.26^***^	0.49^***^	1							
6. T3CSE	0.16^**^	0.20^***^	0.45^***^	0.26^***^	0.30^***^	1						
7. T1PAS	0.46^***^	0.23^***^	0.24^***^	0.28^***^	0.25^***^	0.19^***^	1					
8. T2PAS	0.17^***^	0.39^***^	0.23^***^	0.16^***^	0.30^***^	0.21^***^	0.43^***^	1				
9. T3PAS	0.18^***^	0.21^***^	0.46^***^	0.16^***^	0.14^***^	0.29^***^	0.33^***^	0.36^***^	1			
10. T1TAS	0.46^***^	0.14^**^	0.21^***^	0.18^***^	0.13^***^	0.13^***^	0.32^***^	0.10^*^	0.16^***^	1		
11. T2TAS	0.26^***^	0.47^***^	0.24^***^	0.18^***^	0.30^***^	0.15^***^	0.15^**^	0.41^***^	0.26^***^	0.28^***^	1	
12. T3TAS	0.15^**^	0.20^***^	0.48^***^	0.17^***^	0.17^***^	0.29^***^	0.17^**^	0.18^***^	0.40	0.22^***^	0.26^***^	1
*M*	3.70	3.65	3.81	3.31	3.36	3.68	3.67	3.56	3.79	3.79	3.71	3.78
*SD*	0.53	0.50	0.48	0.65	0.59	0.44	0.61	0.70	0.50	0.58	0.63	0.43

T1 is short for time 1, which is the time when the subject first took the test, and T2 is short for time 2. T3 is short for time 3, the same as below.

PAS is short for parental autonomy support, TAS is short for teacher autonomy support, CSE is short for core self-evaluations, and CA is short for career adaptability, the same as below.

*p < 0.05, **p < 0.01, ***p < 0.001, the same as below.

### 3.3 Model analysis

**Figure 1:**
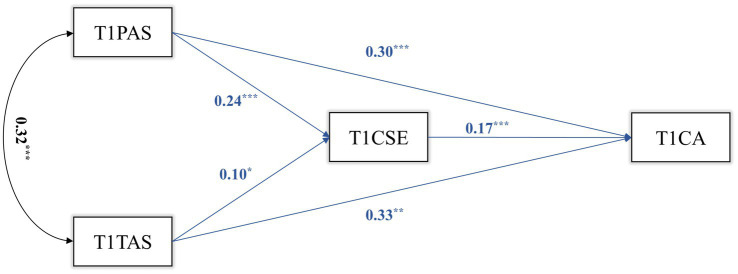
T1 mediation model.

**Figure 2:**
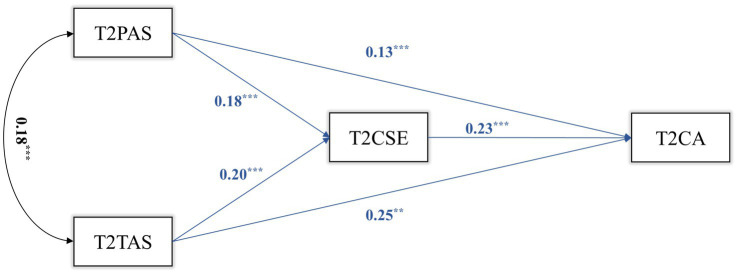
T2 mediation model.

**Figure 3:**
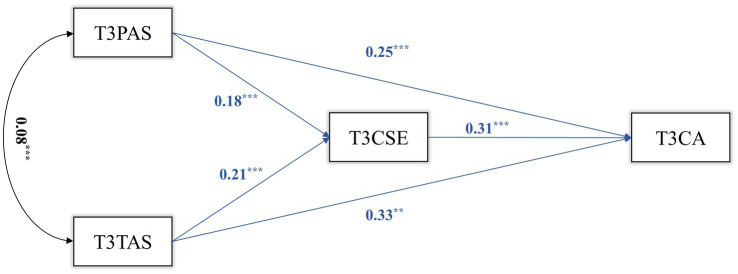
T3 mediation model.

We constructed a mediation model for T1, T2, and T3 transversally. The results are shown in Figures 1–3. In T1, T1CSE had a significant mediating effect between T1PAS and T1CA (ab = 0.037, *p* = 0.002, 95%CI = 0.014, 0.060). The mediating effect of T2CSE between T2TAS and T2CA was not significant (*p* = 0.060). In T2, T2CSE had a significant mediating effect between T2PAS and T2CA (ab = 0.041, *p* = 0.001, 95%CI = 0.017, 0.066). T2CSE had a significant mediating effect between T2TAS and T2CA (ab = 0.046, *p* < 0.001, 95%CI = 0.022, 0.070). In T3, T3CSE had a significant mediating effect between T3PAS and T3CA (ab = 0.059, *p* < 0.001, 95%CI = 0.028, 0.090). T3CSE had a significant mediating effect between T3TAS and T3CA (ab = 0.066, *p* < 0.001, 95%CI = 0.029, 0.104).

The results show that in each cross-sectional study (T1, T2, T3), both parental autonomy support and teacher autonomy support can positively predict middle school students’ career adaptability, and parental autonomy support and teacher autonomy support can also positively predict middle school students’ career adaptability through the mediating role of core self-evaluations.

Subsequently, we controlled for gender and constructed cross-lagged models of career adaptability, core self-evaluations, parental autonomy support, and teacher autonomy support with three-time points. After stepwise removal of insignificant paths, the final cross-lag model fit metrics obtained were as follows: χ2 = 107.778, *df* = 45, χ^2^/*df* = 2.395, RMSEA (90%CI) = 0.054 (0.041, 0.067), CFI = 0.943, TLI = 0.913, SRMR = 0.073.

**Figure 4:**
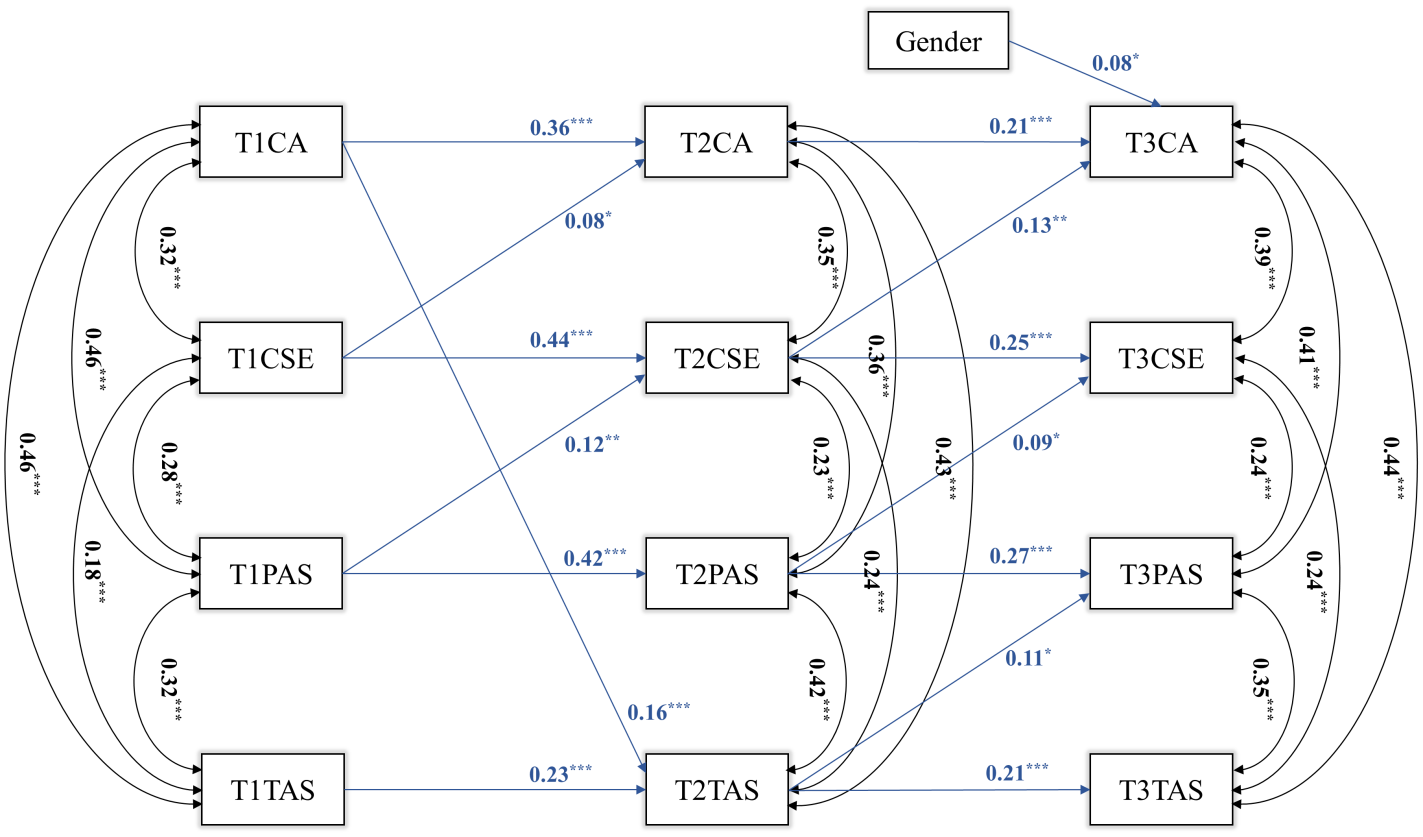
A cross-lag model.

The results in Figure 4 show that from T1 to T2, T1PAS could predict T2CSE (*β* = 0.12, *p* = 0.001), T1CSE could predict T2CA (*β* = 0.08, *p* = 0.047), and T1CA could predict T2TAS (*β* = 0.16, *p* < 0.001). From T2 to T3, T2PAS could predict T3CSE (*β* = 0.09, *p* = 0.026), T2CSE could predict T3CA (*β* = 0.13, *p* = 0.002), and T2TAS could predict T3PAS (*β* = 0.11, *p* = 0.020).

Bootstrap analysis showed that T2CSE had a significant mediating effect between T1PAS and T3CA (ab = 0.015, *p* = 0.022, 95%CI = 0.002, 0.028). The mediating effect of T2TAS between T1CA and T3PAS was not significant (*p* = 0.069).

The results show that T1 parental autonomy support can positively predict T2 core self-evaluations of middle school students, and T2 core self-evaluations can positively predict T3 career adaptability of middle school students. This means that the influence of parental autonomy support on middle school students’ career adaptability through the mediating role of core self-evaluations is directional and stable over time. However, T1 teacher autonomy support did not significantly predict T2 core self-evaluations of middle school students. This means that in the longitudinal relationship, teacher autonomy support cannot influence the career adaptability of middle school students through the mediating effect of core self-evaluations.

## 4 Discussion

### 4.1 Parental and teacher autonomy support enhanced career adaptability

The results indicated a significant positive correlation between parental and teacher autonomy support and career adaptability. Parental and teacher autonomy support positively predicted career adaptability, which confirms H1. This finding not only supports prior research (Guan et al., 2016) but also aligns with the stage-environment matching theory. According to this theory, when external environmental resources can fully meet individuals’ basic psychological needs, it May lead to optimal individual development (Calzo et al., 2020). In other words, the autonomy support provided by parents and teachers helps fulfill the increasing psychological needs of middle school students. The satisfaction of these psychological needs will stimulate individuals’ internal motivation, consequently influencing their career development. Middle school students explore and form preliminary orientations in their career development, which is crucial for establishing values and interests and making career choices. At this stage, the more autonomy support parents and teachers offer to their juveniles, the more beneficial it is for their active exploration and practical experience (Clark and Ladd, 2000), and the better their career adaptability will develop (Öztemel and Yıldız-Akyol, 2021; Xiao et al., 2021). Conversely, the working relationship theory suggests that the absence of appropriate support from parents and teachers, who are important others, at this stage, can impede the career development of children (Kozan et al., 2014).

### 4.2 Core self-evaluations promoted career adaptability

There exists a notable positive correlation between core self-evaluations and career adaptability. T1CSE positively predicts T2CA, and T2CSE positively predicts T3CA. This discovery is corroborated by prior research, which confirms H3. For instance, Zacher (2016) and Xu and Yu (2019) observed in their studies on enterprise employees that core self-evaluations significantly and positively predict the career adaptability of these employees. Similarly, this conclusion extends to the student demographic. Chen et al. (2023) discovered that college students with high core self-evaluations exhibited a greater level of career adaptability and achieved better outcomes in career exploration. According to career construction theory, one of the factors influencing an individual’s career development stems from internal factors, among which personality traits play a crucial role. As a central component of personality, core self-evaluations influence the development of career adaptability (Hirschi et al., 2015). Individuals with higher levels of core self-evaluations tend to provide a more comprehensive and objective assessment. Those with a clearer self-awareness are likely to exhibit greater confidence in navigating career challenges, accomplishing career objectives, and enhancing career adaptability.

### 4.3 Parental and teacher autonomy support strengthened core self-evaluations

The study revealed that parental and teacher autonomy support positively predicted core self-evaluations, which confirms H2. Specifically, T1PAS positively predicted T2CSE, T2PAS positively predicted T3CSE, and parental autonomy support was found to predict core self-evaluations across time in middle school students. Results from the cross-lag analysis indicated that the impact of parental autonomy support was more profound and enduring. This finding is reinforced by previous research. For instance, Bouffard and Labranche (2023) demonstrated that parental autonomy support enhances the self-evaluations of middle school students while reducing school maladjustment. Quested and Duda (2011) explored dance students and observed that those perceiving higher levels of autonomy support exhibited lower social anxiety and body dissatisfaction, along with higher levels of self-esteem and self-evaluations. The underlying reasons for these findings are twofold. First, according to self-determination theory, parents and teachers, as influential figures, can create a supportive environment that nurtures individuality, encourages choice-making, and guides self-exploration in both personal and professional realms. This fosters a more accurate self-understanding, leading to positive self-evaluations (Gagné et al., 2022). Second, middle school students are at a crucial stage of self-awareness development. Support from others during this period, such as fostering independent thinking and identity exploration, prevents individuals from succumbing to extreme distress. Instead, it enables them to view and accept themselves correctly, facilitating objective and rational self-evaluations (Van Petegem et al., 2012).

### 4.4 The mediating role of core self-evaluations

Core self-evaluations partially mediate between teacher and parental autonomy support and career adaptability, which confirms H4. This outcome not only demonstrates the positive influence of core self-evaluations on enhancing career adaptability among middle school students but also elucidates the mechanism by which parental and teacher autonomy support impacts middle school students’ career adaptability. Specifically, parental and teacher autonomy support not only directly affects middle school students’ career adaptability but also influences their career adaptability through core self-evaluations. Career construction theory posits that an individual’s career adaptability arises from the interplay between internal factors and the external environment (Savickas and Porfeli, 2012). This concept aligns with the findings of this study. As external environmental factors, the perceived autonomy support from parents and teachers interacts with core self-evaluations and an internal personality trait and could promote innovation behavior and career development (Ma et al., 2023). Family and school, as pivotal environmental factors, foster a supportive atmosphere for students, encouraging and respecting their choices. The more individuals feel capable and valued, the more confident they become, leading to a more objective and rational self-evaluation. Simultaneously, individuals with a more objective and rational self-awareness are better equipped to confront career development challenges and attain their career goals, thereby improving their career adaptability (Hirschi et al., 2015).

### 4.5 The mediating effect of core self-evaluations across time

Moreover, longitudinal data were employed to evaluate the persistence of the core self-evaluations mediating effect across various time points and investigate the stability of this mediating effect over time. The findings indicated that although teacher autonomy support could forecast middle school students’ core self-evaluations and career adaptability in a cross-sectional study, this association exhibited temporal instability. This variability may be attributed to the specific characteristics of the participants. The subjects in this research cohort are middle school students who May have not yet formed close and enduring bonds with their teachers.

Examining the mediation model from T1 to T3 (Figure 1), it is evident that the predictive impact of teacher autonomy support on the core self-evaluations of middle school students gradually intensifies (*β* = 0.10 to 0.21) over time, signifying an escalating influence of teachers on students as time progresses. A study by Kleinkorres et al. (2023) offers valuable insights. They note that teacher autonomy support is more susceptible to fluctuations compared to parental autonomy support, with school satisfaction and academic performance influencing middle school students’ perceptions of teacher autonomy support. This partially clarifies why teacher autonomy support May not consistently predict middle school students’ core self-evaluations and career adaptability longitudinally. Nonetheless, it remains undisputed that teacher autonomy support can enhance the self-evaluations of middle school students (Quested and Duda, 2011) and empower them to define career objectives and pursue professional success (Lazarová et al., 2019).

T2CSE exhibited a significant mediating effect between T1PAS and T3CA. This model illustrates that robust familial support can foster career advancement by bolstering self-cognitive capabilities. Simply put, individuals’ perceived external emotional support progressively enhances their accurate self-perception over time, empowering them to confront future career challenges and transitions confidently. This assertion is supported by Hou et al. (2019), who maintained that judicious utilization of external support systems aids individuals in overcoming setbacks, developing a correct self-concept, fostering self-assurance, and enhancing adaptability to prospective career environments. Additionally, Liang et al. (2020) conducted a 10-month longitudinal study involving 451 adolescents, revealing that parental supportive behaviors catering to individual autonomy stimulate self-determination awareness, prompting adolescents to contemplate how their present actions influence future career trajectories and strategize toward pursuing their career development goals. Hence, it is crucial for adolescent development that parents validate their children’s autonomous decisions and create an inclusive environment that is conducive to their growth.

Finally, the study was conducted in middle schools in a city in eastern China. Given the objective environment of the subjects in this study, the results are more applicable to urban students in collectivist countries. It is important to note that Chinese adolescents receive significantly lower autonomy support than their North American counterparts (Lekes et al., 2010). While both Chinese and North American adolescents benefit from autonomy support in terms of development and adaptation, the association between autonomy support and career adaptability may be stronger among North American adolescents. Longitudinal studies have shown that while autonomy support is beneficial for both American and Chinese adolescents, the impact on mental function over time is greater in the United States (Wang et al., 2007).

Furthermore, there is an imbalance between urban and rural development in China, with rural development significantly lagging behind urban development (Yin et al., 2022). This makes it challenging to generalize the conclusions of this study to rural students in China. Additionally, there are approximately 9 million left-behind children in rural China who experience neglect and receive less autonomy support, leading to lower career adaptability (Wen et al., 2021). The parental autonomy support of rural students is lower than that of teachers, which May weaken the predictive ability of parental autonomy support on career adaptability across time in longitudinal studies, and the promotion effect of rural students’ parental autonomy support on youth career development is weaker (Sun et al., 2015). In our study, parental autonomy support has a stronger and more stable promoting effect on middle school students’ career adaptability compared with teacher autonomy support. Therefore, when studying the relationship between autonomy support and career adaptability, we should pay attention to the control and analysis of social and cultural factors and economic development levels.

It is evident that many college students express dissatisfaction with their chosen major (Cimsir, 2019) and experience various maladjustment problems (Taylor et al., 2014). This phenomenon is widespread in China, where parents and teachers tend to exert more control over students’ career choices from an early age rather than encouraging autonomy exploration and self-determination (Luo et al., 2013). Consequently, Chinese teenagers May miss out on opportunities for autonomy development, hindering their ability to understand themselves accurately and form clear interests and abilities, causing challenges in navigating their chosen career paths. Therefore, when studying the relationship between autonomy support and career adaptability, it is essential to consider social and cultural factors and economic development levels.

## 5 Conclusion and implications

In conclusion, this study highlights the pivotal role of parental and teacher autonomy support in fostering middle school students’ career adaptability by enhancing their core self-evaluations. The findings reveal a complex interplay between external support systems and internal self-perception, suggesting that nurturing environments created by parents and teachers are essential for promoting positive self-assessment and career adaptability. This relationship emphasizes the importance of fostering autonomy and supportive interactions during critical developmental stages, particularly in the formative years of adolescence.

Moreover, the longitudinal aspect of the study highlights the positive impact of autonomy support on middle school students’ career adaptability, which, in turn, promotes career development by enhancing core self-evaluations. Notably, parental autonomy support appears to have a more stable effect compared to teacher autonomy support. The evolving influence of teacher support over time indicates, that while its immediate effects May fluctuate, its long-term impact strengthens as relationships with students deepen.

This highlights the importance of educators remaining attentive to their role in shaping students’ self-evaluations and influencing their career trajectories.

According to career construction theory, career adaptability is a dynamic mental structure and a malleable cognitive element that can be developed through training, counseling, and education (Coetzee and Harry, 2014). Based on the research findings discussed above, this study offers the following suggestions for enhancing the career adaptability of middle school students, drawing from self-determination theory.

First, it is recommended that career planning courses be introduced as mandatory subjects in middle schools. These courses should include participation from parents or guardians to assist students in discovering the intersection between their interests and career development.

Second, establishing a professional team of career counselors and creating a dedicated career counseling center can be beneficial. This team can provide differentiated career services based on students’ interests and strengths, helping them develop practical career plans tailored to their unique needs.

Finally, fostering a warm, supportive, and understanding family environment is crucial. Parents should avoid excessive interference in their children’s choices and allow them to make autonomous decisions and have personal space. Parents should adopt an observer’s perspective, guiding and encouraging their children to explore their interests and abilities.

## 6 Limitations and prospects

Although this study delves into the link and internal mechanism between parent and teacher autonomy support and the career adaptability of middle school students, several limitations should be noted. The study was conducted in middle schools in a city in eastern China, and due to objective constraints, the sample size was not extensive enough. Therefore, caution should be exercised when generalizing the findings to other countries or regions.

It is important to recognize that China, as a developing country, faces intense social competition and imposes high academic pressure on teenagers (Lu and Chang, 2013). Parents and teachers prioritize academic performance, leaving little room for teenagers to cultivate hobbies. Furthermore, China’s collectivist culture places greater emphasis on the interests of the group, which can, to some extent, limit the development of individual autonomy (Marbell-Pierre et al., 2019). Studies have shown that parental autonomy support for adolescents in the United States and Canada is notably higher than in China (Lekes et al., 2010).

Moreover, there are significant differences in the social and economic status between urban and rural Chinese families, leading to variations in children’s self-determination and core self-evaluations (Ren et al., 2021). The use of self-report scales to measure variables May have been influenced by social approval bias, despite efforts to control for this bias during measurement and data processing. Finally, the study conducted only three follow-up sessions over a 1-year period, which May not fully capture the dynamic changes in career adaptability as middle school students transition through this developmental stage.

Future studies should consider expanding the sample range by employing stratified sampling across multiple regions, including rural and urban areas, as well as countries with diverse cultural orientations, to address these limitations. Longitudinal studies could benefit from a larger sample size, increased data collection waves, and a broader age range to explore youth career development from middle school through university. To reduce the influence of social approval bias, researchers could incorporate various methods, such as interviews or case studies, to enhance the comprehensiveness of the research.

Moreover, extending the duration of tracking and increasing the number of measurements in future studies would provide a more comprehensive understanding of the developmental trajectory of middle school students’ career adaptability and the causal relationships between its variables. Finally, further research is needed to explore career counseling approaches that apply theoretical findings on career adaptability to the practical promotion of adolescent career development.

## Data Availability

The raw data supporting the conclusions of this article will be made available by the authors, without undue reservation.
